# Immune recognition of syngeneic, allogeneic and xenogeneic stromal cell transplants in healthy retinas

**DOI:** 10.1186/s13287-022-03129-y

**Published:** 2022-08-20

**Authors:** María Norte-Muñoz, Alejandro Gallego-Ortega, Fernando Lucas-Ruiz, María J. González-Riquelme, Yazmín I. Changa-Espinoza, Caridad Galindo-Romero, Peter Ponsaerts, Manuel Vidal-Sanz, David García-Bernal, Marta Agudo-Barriuso

**Affiliations:** 1 Experimental Ophthalmology Group, Instituto Murciano de Investigación Biosanitaria Virgen de la Arrixaca (IMIB-Arrixaca) & Universidad de Murcia, Murcia, Spain; 2 Laboratory of Experimental Hematology, Vaccine and Infectious Disease Institute (Vaxinfectio), University of Antwerp, Antwerp, Belgium; 3 Hematopoietic Transplant and Cellular Therapy Unit, Instituto Murciano de Investigación Biosanitaria Virgen de la Arrixaca (IMIB-Arrixaca) & Biochemistry, Molecular Biology and Immunology Department, Universidad de Murcia, Murcia, Spain

**Keywords:** Müller cells, CD45, ERG, Retinal ganglion cells, BM-MSCs

## Abstract

**Background:**

Advanced therapies using adult mesenchymal stromal cells (MSCs) for neurodegenerative diseases are not effectively translated into the clinic. The cross talk between the transplanted cells and the host tissue is something that, despite its importance, is not being systematically investigated.

**Methods:**

We have compared the response of the mouse healthy retina to the intravitreal transplantation of MSCs derived from the bone marrow in four modalities: syngeneic, allogeneic, xenogeneic and allogeneic with immunosuppression using functional analysis in vivo and histology, cytometry and protein measurement *post-mortem*. Data were considered significant (*p* < 0.05) after nonparametric suitable statistical tests.

**Results:**

Transplanted cells remain in the vitreous and are cleared by microglial cells a process that is quicker in allotransplants regardless of immunosuppression. All transplants cause anatomical remodelling which is more severe after xenotransplants. Xeno- and allotransplants with or without immunosuppression cause macro- and microglial activation and retinal functional impairment, being xenotransplants the most detrimental and the only ones that recruit CD45^+^Iba1^−^cells. The profile of proinflammatory cytokines changes in all transplantation settings. However, none of these changes affect the retinal ganglion cell population.

**Conclusions:**

We show here a specific functional and anatomical retinal response depending on the MSC transplantation modality, an aspect that should be taken into consideration when conducting preclinical studies if we intend a more realistic translation into clinical practice.

**Supplementary Information:**

The online version contains supplementary material available at 10.1186/s13287-022-03129-y.

## Background

Neurodegenerative diseases lead to a permanent loss of cognitive, sensory or motor function. They are caused by a multitude of aetiologies, the type of neurons affected is different and so is the functional impairment. Finding a cure for these diseases is extremely difficult because neither neuronal replacement nor rewiring of lost connections is yet possible. However, neuronal loss can be delayed and neurons in the early steps of degeneration can be rescued using the so-called neuroprotective therapies.

Neuroprotective therapies encompass several strategies from administration of single molecules such as trophic factors targeting survival pathways [1–7] or small antagonists targeting apoptotic or inflammatory pathways [7–12] to cell therapy [13–19].

Stem cell-based therapy is a promising neuroprotective avenue since this heterogeneous population of cells are living bioreactors that, once infiltrated into the affected tissues, are capable of directly secreting via a “hit-and-run” mechanism or stimulating the endogenous secretion of many molecules (*i.e.,* anti-inflammatory molecules and growth factors) which enhance the possibility of tackling several neurodegenerative processes at once. Among all the modalities of advances therapies, mesenchymal stromal cells (MSCs) currently constitute the most frequently used cell type: first, they are isolated without much discomfort from several niches of perinatal or adult tissues circumventing the ethical concerns of embryonic stem cells and second, they possess potent anti-inflammatory and immunomodulatory properties and the capacity to secrete a variety of trophic factors [20]. Furthermore, MSCs tailor their secretome depending on the host microenvironment [21–23]. Nevertheless, and contrary to the previously reported low immunogenicity of MSCs and their ability to avoid being rejected by the host's immune system, more recent studies have suggested that these cells may not be as intrinsically immune privileged as initially thought [24, 25].

Whatever the primary cause of neurodegeneration, it always triggers a series of intertwined pathological processes that amplify the damage, namely neuroinflammation and trophic factor withdrawal which in turn cause oxidative stress, DNA damage, apoptosis and necrosis. These are, therefore, the main targets to achieve neuroprotection and the reason why MSCs are so well placed as therapeutics.

Many preclinical studies on MSC neuroprotective properties have shown promising results [14, 18, 26, 27]. However, there is a lack of concordance between the extensive research on MSC neuroprotection and clinical translation as evidenced by the few clinical trials currently using this strategy (clinicaltrials.gov). This discordance could be due to the lack of systematic preclinical studies that vary in models, MSC source and assays*,* together with the inherent complexity of neurodegenerative diseases.

To bridge the gap between preclinical and clinical studies, it is important to isolate the factors that may affect the success of therapeutic interventions and for that systematic and comparative preclinical studies must be carried out. A major discordance between animal models and patients is the type of transplant. Commonly, human cells are tested in animal models, but this is a xenotransplant which is not the standard clinical practice except for the very novel use of transgenic pigs as donors (first transplant of a modified pig’s heart to a human. Pig engineered by Revivicor, transplant done in the University of Maryland Medical Center 2022). As a rule, though, patients are either treated with autologous (syngeneic) or allogeneic cells. In addition, patients receiving allotransplants undergo immunosuppression to prevent graft rejection, a practice that is not always used in animal models, even when receiving human cells. The effect of the transplant is essential because the host immune response may radically change the therapeutic outcome [18, 28], graft survival, tissue homeostasis and immune response to the graft.

We have recently reported, in a very well-established model of axonal damage and neuronal death [10, 29–31] that the type of transplant changes the neuroprotective and axonal regenerative outcome mediated by bone marrow mesenchymal stromal cells (BM-MSCs) [18]. In that work, we tested MSCs from the same niche in the same model of neurodegeneration in the three transplantation settings, thus isolating the effect of the transplant on neuronal survival and axonal regeneration.

Here we follow up that work and show the differential response of the healthy retina to syngeneic, allogeneic and xenogeneic transplantation of BM-MSCs. Again, we have maintained the tissue (the retina), and the cell type and dose. To compare better to the clinic, we have also studied allotransplants with systemic immunosuppression.

The mouse retina offers several advantages over other parts of the CNS: (i) it is easily accessible and treatments can be administered in the vitreous chamber that bathes the retina, (ii) due to the transparency of the anterior part of the eye, the retina can be studied anatomically and functionally in vivo with non-invasive techniques, such as optical coherence tomography (OCT) and full-field electroretinogram (ERG), respectively, and (iii) in post-mortem tissue it is possible to assess very precisely anatomical (i.e., gliosis, neuronal death, structural abnormalities) and molecular changes.

After intravitreal administration of BM-MSCs in these settings: syngeneic (mouse BM-MSCs from C57BL/6 mouse strain to C57BL/6 mice), allogeneic (mouse BM-MSCs from C57BL/6 mice to BALB/c mice), xenogeneic (human BMSCs to C57BL/6 mice) and allogeneic with systemic immunosuppression, we have studied from 3 to 21 days after transplantation the retinal integrity anatomically and functionally, the glial response, the effect on the profile of pro- and anti-inflammatory mediators and whether the transplants cause neuronal loss.

## Methods

### Animal handling

All animal procedures were approved by the Institutional Animal Care and Use Committee at University of Murcia (Murcia, Spain) and performed according to the guidelines of our Institution (approved protocols A13150201 and A1320140704).

Two-month-old male mice (C57BL/6, BALB/c and C57BL/6-Tg (CAG-EGFP) strains) were obtained from the breeding colony of the University of Murcia or purchased from Envigo (Barcelona, Spain). Animals were kept at the University of Murcia animal housing facilities in temperature- and light-controlled rooms (12 h light/dark cycles) with food and water administered ad libitum.

All in vivo analyses were carried out under general anaesthesia administered intraperitoneally with a mixture of ketamine (60 mg/kg, Ketolar, Parke-Davies, S.L., Barcelona, Spain) and xylazine (10 mg/kg, Rompún, Bayer S.A., Barcelona, Spain). Analgesia was provided by subcutaneous administration of buprenorphine (0.1 mg/kg; Buprex, Buprenorphine 0.3 mg/mL; Schering-Plough, Madrid, Spain). During and after anaesthesia, eyes were covered with an ointment (Tobrex; Alcon S.A., Barcelona, Spain) to prevent corneal desiccation. Animals were killed with an intraperitoneal injection of an overdose of sodium pentobarbital (Dolethal, Vetoquinol; Especialidades Veterinarias, S.A., Alcobendas, Madrid, Spain).

Immunosupression was carried out following approved protocol by University of Murcia (A13191105) that combines intraperitoneal daily dexamethasone injection (1.6 mg/kg; Cortexonavet 2 mg/mL; Syva, León, Spain) and oral cyclosporine (210 mg/L; Cyclavance® 100 mg/mL; Virbac, Barcelona, Spain) diluted in water.

### Experimental design

**Fig. 1:**
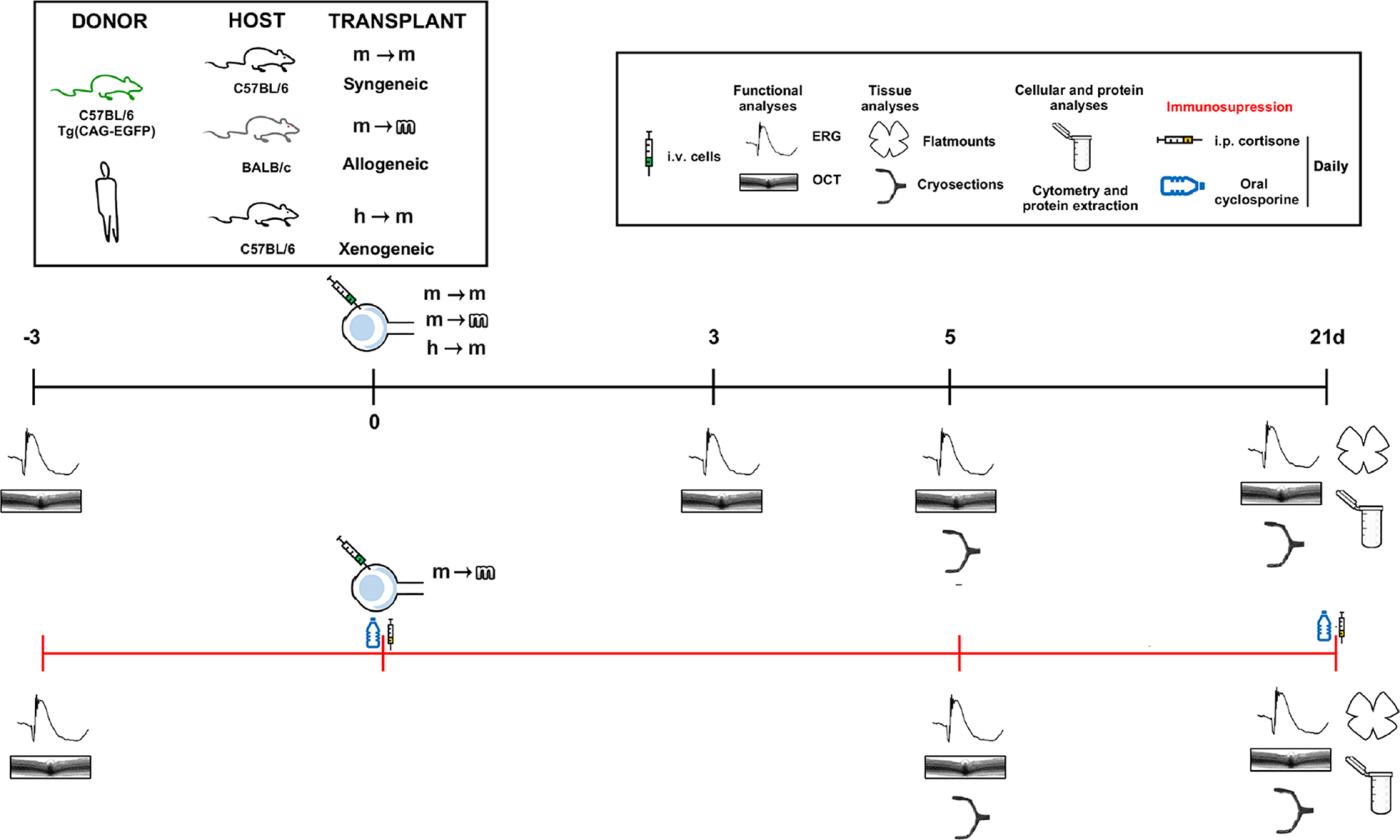
Experimental design. Black temporal line, no immunosuppression. Red temporal line, immunosuppression. *n* = 4–6 per assay, group and time point

Figure 1 summarizes the experimental groups and analyses. In vivo functional and anatomical analyses (OCT and ERG) were assessed longitudinally before (pre- or baseline) and after the procedures in non-immunosuppressed animals. Immunosuppressed animals were divided in two groups, one analysed at 5 days and the other at 21 days. Intact animals were used as control for the total number of retinal ganglion cells (RGCs) and resting glial and cytokine secretion activities.

### Isolation, culture and characterization of human and mouse bone marrow mesenchymal stem cells

To obtain human mesenchymal stromal cells, human bone marrow samples (hBM) were obtained by direct percutaneous aspiration from the iliac crest of 6 healthy volunteers at Hospital Clinico Universitario Virgen de la Arrixaca (Murcia, Spain). All protocols used to obtain and process human samples were approved by the local ethics committees (HUSA19/1531.17/02/2020) according to Spanish and European legislation and conformed to the ethical guidelines of the Helsinki Declaration. Donors provided written informed consent before obtaining samples. Bone marrow was collected in syringes containing 20 U/ml sodium heparin and mononuclear cell fraction collected after a Ficoll-Paque density gradient separation by centrifugation following previously described method. After that, mononuclear cell fraction was rinsed twice with phosphate-buffered saline (PBS) (Merck Life Science S.L.U., Madrid, Spain) and seeded into 75-cm^2^ culture flasks (Merck Life Science) at a density of 1.6 × 10^5^ cells/cm^2^ in Minimum Essential Medium Eagle (Thermo Fisher Scientific, Madrid, Spain) containing 10% foetal bovine serum (FBS) (BioWhittaker, Walkersville, MA, USA), 1% penicillin/streptomycin (P/S) (Thermo Fisher Scientific, Madrid Spain) and 1% GlutaMAX™ (Thermo Fisher Scientific). After 3 days of undisturbed cultures at 37 °C and 5% CO_2_, unattached cells were removed and fresh culture medium was added and replaced twice a week. Mesenchymal phenotype of hBM-MSCs was verified by flow cytometry using specific antibodies for human CD73, CD90, CD105, CD14, CD20, CD34 and CD45 (human MSC phenotyping cocktail; Miltenyi Biotec, Bergisch Gladbach, Germany) in a FACS Canto flow cytometer (Becton Dickinson, Franklin Lakes, NJ, USA) as previously reported [18] (Additional file 1: Fig. S1A, left).

Mouse bone marrow mesenchymal stromal cells (mBM-MSCs) were isolated from β-actin-GFP transgenic C57BL/6-Tg (CAG-EGFP) mice. Tibias and femurs were collected from 6 to 8 weeks mice, bone epiphyses were excised, and bone marrow was flushed out using a 25-gauge needle and syringe containing low-glucose Dulbecco's modified Eagle's medium (DMEM) (Thermo Fisher Scientific). After that, cells were seeded into 75-cm^2^ culture flasks at a density of 1.6 × 10^5^ cells/cm^2^ and cultured in low-glucose DMEM medium containing 15% FBS, 1% P/S and 1% GlutaMAX™ following the same protocol as for human cells. Mesenchymal phenotype of mBM-MSCs was verified by flow cytometry as above using specific antibodies for mouse CD73, CD90, CD105, CD34 and CD45 (all from BioLegend, San Diego, CA, USA) as previously reported [18] (Additional file 1: Fig. S1A, right).

To analyse multipotent differentiation potential of human and mouse BM-MSCs, cells were cultured in StemMACS™ AdipoDiff, OsteoDiff and ChondroDiff Media for human MSCs (Miltenyi Biotec) and mouse MSC functional identification kit (R&D Systems, Minneapolis, MN, USA), respectively, following the manufacturer’s instructions. For assessment of adipogenic differentiation, cells were cultured for 14 days, washed, fixed with cold 70% methanol for 5 min and stained with Oil Red O for detecting cytoplasmic lipid droplets. For analysis of osteogenic differentiation, cells were cultured for 21 days, fixed with 4% paraformaldehyde in PBS and stained with BCIP/NBT (Sigma-Aldrich, St. Louis, MO, USA) or Alizarin Red (Sigma-Aldrich) to detect alkaline phosphatase activity or calcium deposition, respectively. For evaluating chondrogenic differentiation, cells were centrifuged to obtain a pellet, cultured for 21 days in a 15-mL polypropylene tube, fixed with 4% paraformaldehyde in PBS, dehydrated and embedded in paraffin and 5-mm-thick sections were cut. Finally, sections were stained with Alcian blue (Sigma-Aldrich) to detect glycosaminoglycan expression and counterstained with eosin (Sigma-Aldrich) (Additional file 1: Fig. S1B).

Apart from the immunophenotype and multipotential differentiation capacity of human and mouse BM-MSCs, we previously performed other control quality analyses of the isolated cells including potency assays (i.e.*,* inhibition of proliferation of phytohaemagglutinin-stimulated human T cells or concanavalin A-stimulated splenocytes, respectively, by BrdU incorporation assays). BM-MSCs isolated from all healthy donors and mice displayed adequate immunosuppressive properties and at comparable levels between samples (not shown).

### Intravitreal injection

Cells were administered in the left eye in DMEM medium at a concentration 8 × 10^3^ cells/µL in 2.5 μL final volume. Intravitreal injections were done following previously published methods [7, 11, 32].

### Flow cytometry

Retinas were collected in neurobasal medium (Thermo Fisher Scientific) supplemented with 10% FBS (Thermo Fisher Scientific), 2% B-27 (Thermo Fisher Scientific) and 1% L-glutamine (Merck Life Science) after CO_2_ euthanasia and dissected mechanically with scalpel. After resuspending up and down twice with micropipette to improve cell dissociation, 0.2% collagenase A (0.223 U/mg; Roche Diagnostics GmbH, Mannheim, Germany) in DMEM was added and incubated for 30 min at 37 °C. After that, cellular suspensions were filtered through a 70-μm Corning™ cell strainer (Thermo Fisher Scientific) and immediately centrifuged for 5 min at 600*g*. Cell pellets were resuspended in complete DMEM medium and primary fluorescence-labelled antibodies were added (1:250 anti-mouse CD11b-FITC; eBioscience, Thermo Fisher Scientific; and 1:500 anti-mouse CD45-PE; eBioscience, Thermo Fisher Scientific). After incubation for 30 min at 4 °C, two washing steps were performed and finally acquired in a FACS Canto flow cytometer. Flow cytometry data were analysed with FlowJo software (FlowJo LLC, Ashland, OR, USA) at the Tissue Culture Facility (ACTI, University of Murcia and IMIB-Arrixaca).

### Protein extraction and ELISA assay

Fresh dissected retinas were immediately submerged in Pro-Prep™ (Intron Biotechnology, Seongram, South Korea) after CO_2_ euthanasia and crushed with a hand shredder. After 2 h, Eppendorf tubes were centrifuged for 15 min at 13,000 r.p.m. Finally, supernatants were collected and stored at − 80 °C until analysed by ELISA.

Each cytokine was measured individually following manufacturer´s instructions using murine TNF-α ELISA kit (Diaclone, Besancon Cedex, France), murine IL-6 ELISA kit (Diaclone), murine TGF-β ELISA kit (Diaclone SAS) and murine PGE2 ELISA kit (R&D Systems, Minneapolis, MN, USA). Absorbances were measured at 450 nm in a spectrophotometer and concentrations calculated from standard curve.

### Electroretinography (ERG)

Full-field ERG was performed as previous published [6, 12, 18, 33]. In brief, after 12 h of dark adaptation, mice were anaesthetized and both eyes dilated with topical mydriatic (Tropicamida 1%; Alcon-Cusí, S.A. Barcelona, Spain). Scotopic and photopic responses were recorded using Burian–Allen corneal bipolar electrodes simultaneously in both eyes. A drop of methylcellulose (Methocel® 2%; Novartis Laboratories CIBA Vision, Annonay, France) was used between the cornea and the electrodes to improve signal conductivity. The reference electrode was placed in the mouth and a needle at the base of the tail was used as a ground electrode. RGC-mediated response was recorded with light flashes ranging from − 4.4 log cd·s/m^2^ scotopically. Rod-mediated response was recorded at − 2.5 log cd·s/m^2^. Mixed (a- and b-waves) response were recorded at − 0.5 log cd·s/m^2^. For cone-mediated response, 0.5 log cd·s/m^2^ flashed were applied on a 30 cd/m^2^ rod-saturated background. The electrical signals were digitized at 20 kHz using a Power Lab data acquisition board (AD Instruments, Chalgrove, UK). Standard ERG waves were analysed according to the International Society for Clinical Electrophysiology of Vision (ISCEV).

### Optical coherence tomography (OCT)

Both retinas were analysed longitudinally under SD-OCT (Spectralis; Heidelberg Engineering, Heidelberg, Germany) adapted with a commercially available 78-D double aspheric fundus lens (Volk Optical, Inc., Mentor, OH, USA) mounted in front of the camera unit as described [6]. After anaesthesia, a drop of tropicamide (Tropicamida 1%; Alcon-Cusí, S.A. Barcelona, Spain) was instilled in both eyes to induce mydriasis. Imaging was performed with a proprietary software package (Eye Explorer, version 3.2.1.0; Heidelberg Engineering). Retinas were imaged using a raster scan of 31 equally spaced horizontal B-scan. Thickness of the total, inner and outer retina was measured manually close to the optic nerve head and at 1-mm from it always in central sections spanning the optic disc. Volume of the central retina was calculated by the software after manually aligning the inner and outer retinal limits.

### Tissue processing and immunodetection

Animals were perfused transcardially with 0.9% saline solution followed by 4% paraformaldehyde in 0.1 M phosphate buffer. Flat mounted retinas were prepared as described [31]. Eyes for cross section were submerged in increasing concentrations of sucrose and embedded in Tissue-Tek® O.C.T. compound (Sakura-Finetek, Barcelona, Spain) and then stored at − 80 °C until being cryostated at 16-µm as described [34].

Immunodetection in flat mounts or cross sections was carried out as previously described [35, 36]. Flat-mounted retinas were immunodetected with mouse anti-Brn3a (1:500; MAB1585, Merck Millipore; Madrid, Spain) to quantify the total number of RGCs. Retinal cross sections were used to follow graft survival and glial activation.

mBM-MSCs were identified by their GFP expression and hBM-MSCs by immunodetection with mouse anti-human mitochondria antibody (1:800, ab3298 Abcam, Cambridge, UK). Glial response was assessed with: rabbit anti-Iba1 (1:500, ab178846 Abcam, Cambridge, UK), goat anti-Vimentin (1:750, sc-7557 Santa Cruz Biotechnology Inc. Heidelberg, Germany), and rat anti-CD45 antibodies (1:500, 30/FN eBioscience, Thermo Fisher Scientific). Secondary detection was carried out with Alexa Fluor-labelled secondary antibodies (1:500; Molecular Probes; Thermo Fisher Scientific, Madrid, Spain). Retinal whole mounts were mounted with anti-fading mounting media and cross sections with the same medium containing DAPI to counterstain all cell nuclei (H-1200, Vectashield®, Vector Laboratories Inc., Burlingame, CA, USA).

### Image acquisition and analyses

Images were acquired using a Leica DM6B epifluorescence microscope (Leica, Wetzlar, Germany). Retinal photomontages were reconstructed from individual squared images of 500 µm^2^. Brn3a^+^RGCs were quantified automatically and their topographical distribution was assessed by isodensity or neighbour maps using previously reported methods [32]. Isodensity maps show the density of RGCs with a colour scale that goes from 0–500 RGCs/mm^2^ (purple) to ≥ 3200 RGCs/mm^2^ (red).

GFP^+^ or human mitochondrial^+^ BM-MSCs and Iba1^+^ cells in the vitreous body were quantified in the same images (3 sections per animal, *n* = 4 animals/group/time point). Vimentin signal intensity along each section was measured using ImageJ software (developed by Wayne Rasband, National Institutes of Health, Bethesda, MD, USA; https://imagej.nih.gov/ij, 13th January 2022). Fluorescence in intact retinas was used as 100% value (3 sections per animal, *n* = 4 animals/group/time point).

### Statistical analyses

Data were analysed and plotted with GraphPad Prism v.7 (GraphPad Software, San Diego, CA, USA) and presented as mean ± standard deviation (SD). Differences were considered significant when *p* < 0.05. Statistical tests are detailed in results.

## Results

### Allotransplants and xenotransplants alter the retinal functionality

**Fig. 2:**
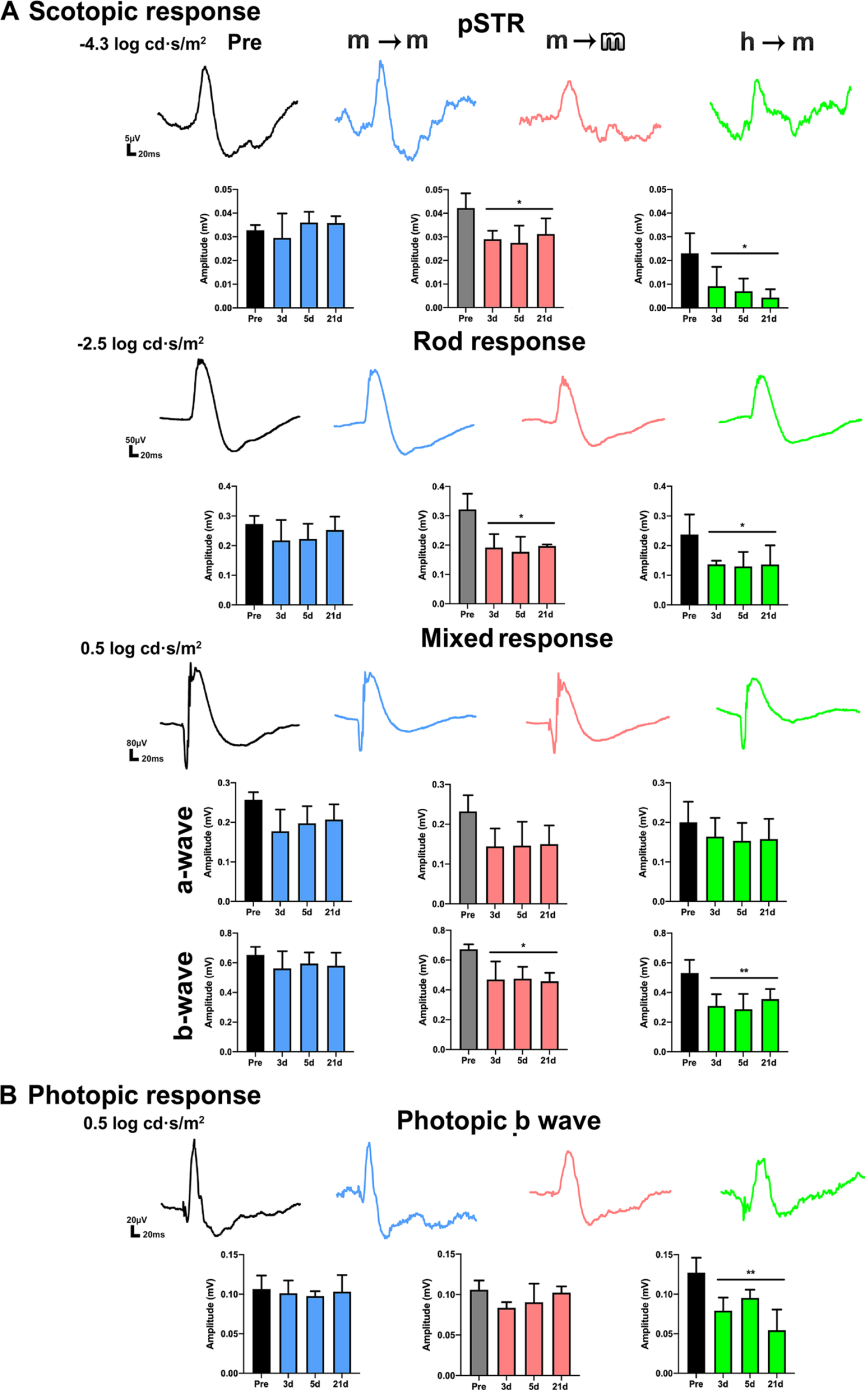
Functional impairment in allo- and xenotransplanted retinas. Electroretinogram waves and column graphs (mean wave amplitude ± SD) obtained longitudinally from the scotopic (**A**) and photopic (**B**) responses at the different light pulses before (pre-: black) and at 3, 5 and 21 days after each transplant (blue: syngeneic; pink: allogeneic; green: xenogeneic). pSTR: RGCs; scotopic rod response: rod bipolar cells; scotopic mixed response: a-wave: cones and rods, b-wave: cone and rod bipolar cells; photopic b-wave: cone bipolar cells. *Significant compared to baseline values (*p < 0.05, **p < 0.01, nonparametric, paired Friedman test and Dunn’s post hoc analysis). *n* = 6 animals/group

Retinal functionality was measured by full-field electroretinogram in the same animals before (pre-) and at 3, 5 and 21 days after intravitreal cell transplant. Already 3 days after the administration of BM-MSCs and up to 21 days all scotopic waves, except the a-wave from the mixed response, were significantly decreased in the allo- and xenotransplanted retinas remaining within baseline values in the syngeneic group (Fig. 2A). The affected scotopic waves reflect an alteration in the function of: (i) RGCs (pSTR) which in per cent was significantly more affected in xeno- (decrease of 85% ± 9) than in allotransplants (25% ± 14); (ii) rod bipolar cells (rod response); and (iii) cone and rod bipolar cells (b-wave from the mixed response). Under photopic conditions, neither syngeneic nor allogeneic transplants had a significant effect, but the xenotransplant decreased the cone bipolar cells response at all time points (Fig. 2B).

### All transplants induce anatomical changes in the retina, with the greatest retinal alteration observed after xenotransplantation

**Fig. 3:**
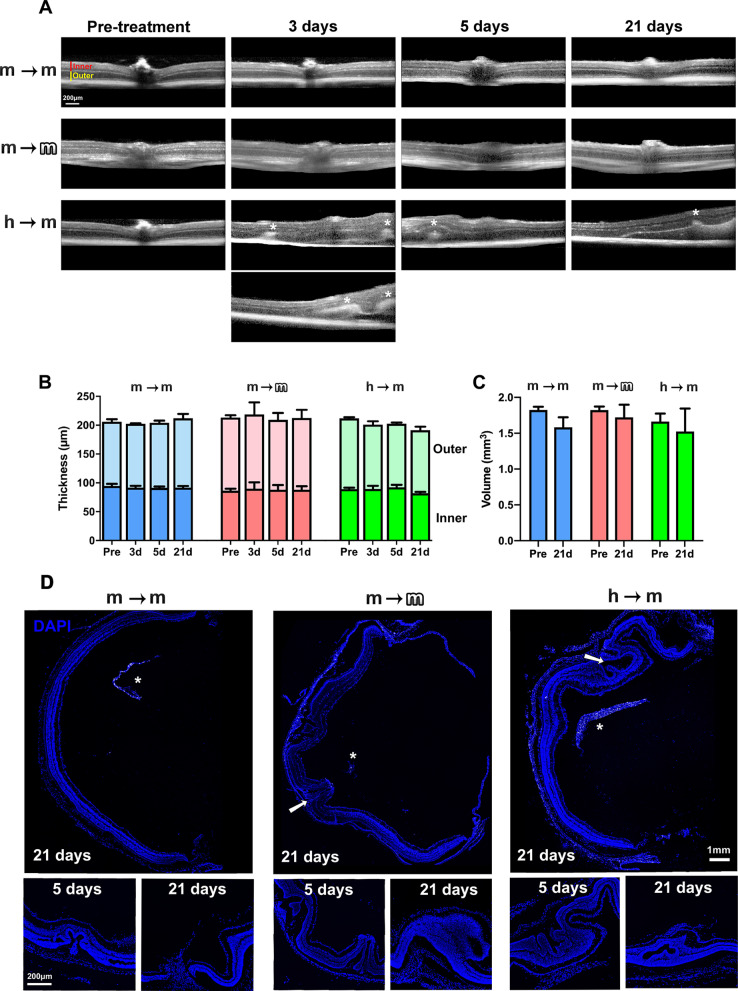
BM-MSCs transplants alter the retinal architecture. **A** OCT sections spanning the optic nerve were taken longitudinally before and 3, 5 and 21 days after each transplant. Asterisks mark areas of hyperrefringence, retinal folding and detachment. **B** Stacked column graphs showing the total, outer and inner retinal thickness were measured in the OCT images at 1-mm from the optic nerve (mean ± SD) before and at 3, 5 and 21 days after each transplant. **C** Column graph showing the central retinal volume before and 21 days after each transplant. There were not significant differences between groups (*p* > 0.05, nonparametric, paired Friedman test and Dunn’s post hoc analysis). *n* = 6 animals/group. **D** DAPI-stained retinal sections from transplanted animals (for intact animals see Additional file 2: Fig. S2A). In all transplants, the retina shows structural alterations such as folds (white arrows), holes and detachment already at 5 days post-transplant. Asterisks mark the BM-MSC mesh in the vitreous. *n* = 4 retinas/group/timepoint

First, we imaged and measured the retina in live animals using SD-OCT before and at 3, 5 and 21 days after transplantation. As shown in Fig. 3A, the retinal structure appears normal in the syngeneic and allogeneic groups. However, in the xenotransplanted retinas there were hyperreflective spots, visible from 3 days, and retinal folds observed at 21 days. These changes did not modify the retinal thickness measured at 1 mm from the optic nerve, nor the volume of the central retina (Fig. 3B, C).

When we examined the retinal cross-sectional *post-mortem*, there were signs of retinal remodelling in the three transplants: anomalous growths, folds and retinal detachment. These alterations were generally located in the areas closest to the mesh that the BM-MSCs formed the vitreous after administration (see below). Thus, the abnormalities could be peripheral or central depending on where the cells were in the vitreous (Fig. 3D). To ascertain that retinal folds were not caused by the injection itself, we injected 4 eyes with vehicle and analysed the animals 5 days post-injection. All retinas showed a normal architecture (Additional file 2: Fig. S2A).

These architectural anomalies were more frequent in xenotransplants (100% of retinas) than in allo- or syngeneic transplants (25%). In addition, they were more developed at 21 days than at 5 and more dramatic in xeno- than in allotransplanted retinas and in these than in syngeneically transplanted ones.

### Müller cell hypertrophy in allo- and xenotransplanted retinas

**Fig. 4:**
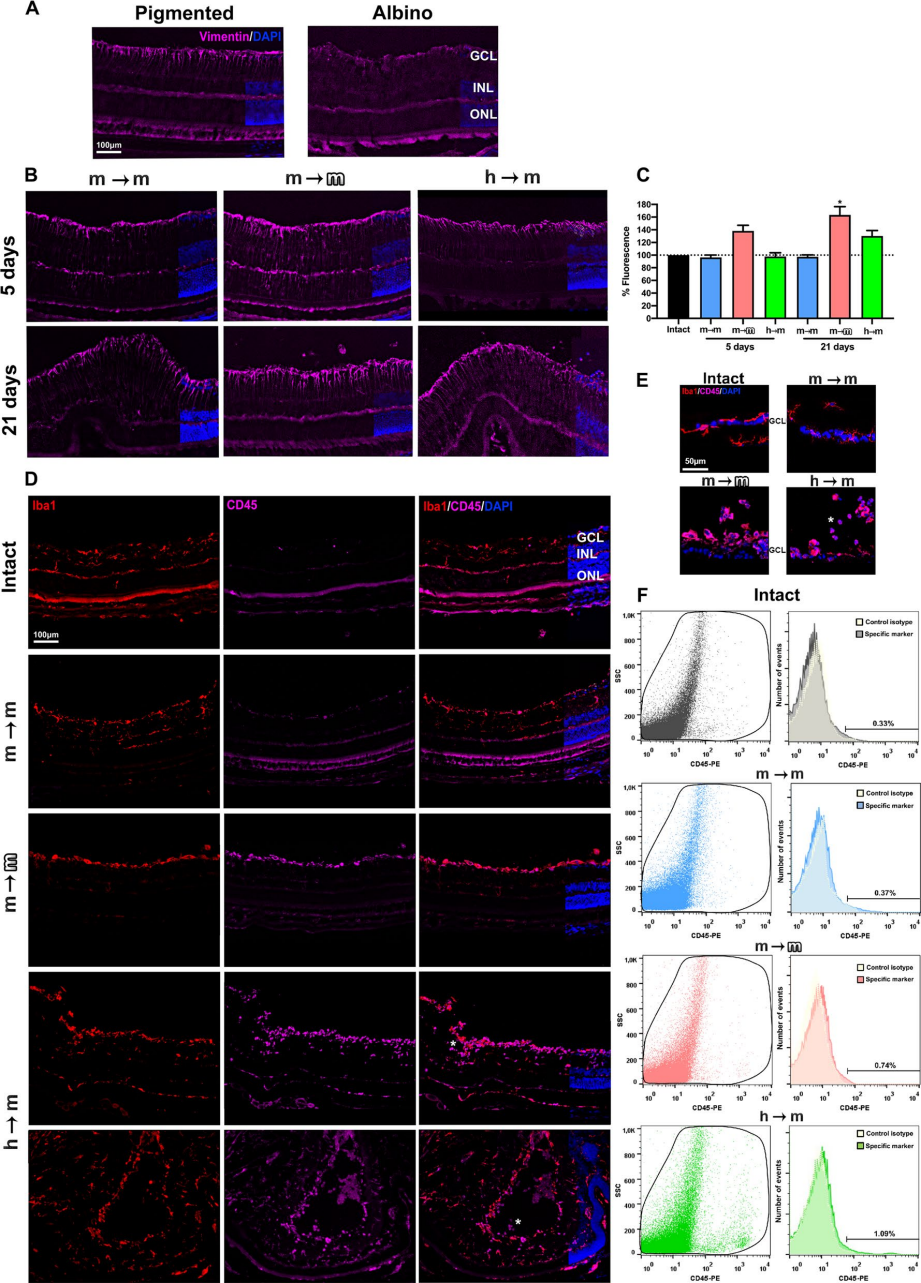
BM-MSC transplants trigger Müller cell hypertrophy, microglial activation and recruitment of CD45^+^ cells. **A:** Retinal magnifications from intact pigmented and albino animals showing the normal expression of vimentin in Müller cells (purple). **B:** Vimentin expression (purple) in transplanted retinas at 5 and 21 days. **C**: Column graph showing the signal intensity of vimentin relative to intact (mean signal in albino and pigmented, 100% arbitrary units) in all transplanted retinas at both time points (*n* = 4 retinas/group/timepoint, 3 sections/retina). Müller cell hypertrophy was significant in allotransplanted retinas at 5 and 21 days and in xenotransplanted retinas at 21 days (*p < 0.05 compared to intact, nonparametric Mann–Whitney *t* test). **D:** Retinal magnifications showing the normal distribution of microglial cells (Iba1^+^, red) and CD45^+^ cells (purple) in intact and 21 days trasnplanted retinas. Asterisks mark CD45^+^ Iba1^−^ cells. **E**: Flow cytometry histograms and dot plots showing the per cent of CD45^+^ cells in intact and transplanted retinas. In xenotransplanted and allotransplanted retinas, the per cent of CD45^+^ cells was much higher than in intact or in syngeneic transplant. **F**: Retinal magnifications from intact and transplanted groups showing the morphology of microglial cells (Iba1^+^, red), CD45^+^ cells (purple) and their co-localization with DAPI staining. *n* = 4 retinas/group/assay

Müller cells are the radial glia of the retina. These cells respond to injury and stress by upregulating the expression of intermediate filaments such as vimentin. We measured the expression of vimentin by immunofluorescence in cross sections including healthy and remodelled areas of the retina. In the latter, Müller cells were gliotic in all transplants. Considering the whole cross sections, we observed that syngeneic transplants did not cause a significant gliosis, while there was a significant hypertrophy at 5 days in allotransplants and at 21 days in allo- and xenotransplants (Fig. 4A, B).

### Microglial activation and recruitment of CD45^+^ cells in the transplanted retinas

Next, we studied microglial cell response 21 days after the grafts transplants using Iba1 and CD45 immunodetection to visualize them in their resting and activated states, respectively. In all graftstransplants, microglial cells were activated in the areas of retinal abnormalities. In the retinal areas without remodelling Iba1 and CD45 staining in intact and syngeneically transplanted retinas was very similar and microglial cells had a resting morphology with small somas and thin long branches. In the allotransplanted retinas, Iba1 and CD45 staining was brighter than in intact ones and co-localized in most cells. The higher expression of CD45 by Iba1^+^ microglia indicates that they were activated in accordance with their rounded morphology (Fig. 4D, E). Interestingly, they were mostly localized in the innermost layer of the retina, the nerve fibre layer, closest to the vitreous (Fig. 4F and Additional file 2: Fig. S2B). In xenotransplanted retinas, in addition to those features observed in allotransplants, there were many CD45^+^ Iba1^−^ cells in all retinal layers although they were more abundant in the ganglion cell and inner plexiform layers (Fig. 4D).

To know whether BM-MSC transplant altered the number of CD45^+^ cells, we performed flow cytometry analyses. In intact and syngeneically transplanted retinas, the percentage of CD45^+^ cells was similar (0.33%, 0.37%), while it doubled (0.7%) after allotransplant, and tripled (1.09%) after xenotransplant (Fig. 4F).

Thus, these data indicate that the syngeneic transplant does not cause a significant microglial activation as the allo- and xenotransplant do. Allo- and xenotransplants activate microglial cells, and xenotransplants also recruit CD45^+^ cells.

### Microglial cells migrate to the vitreous and surround the BM-MSCs

BM-MSCs did not integrate into the retina, but were observed in the vitreous body, and over the retina forming a meshwork as previously reported [18] (Fig. 3).

**Fig. 5:**
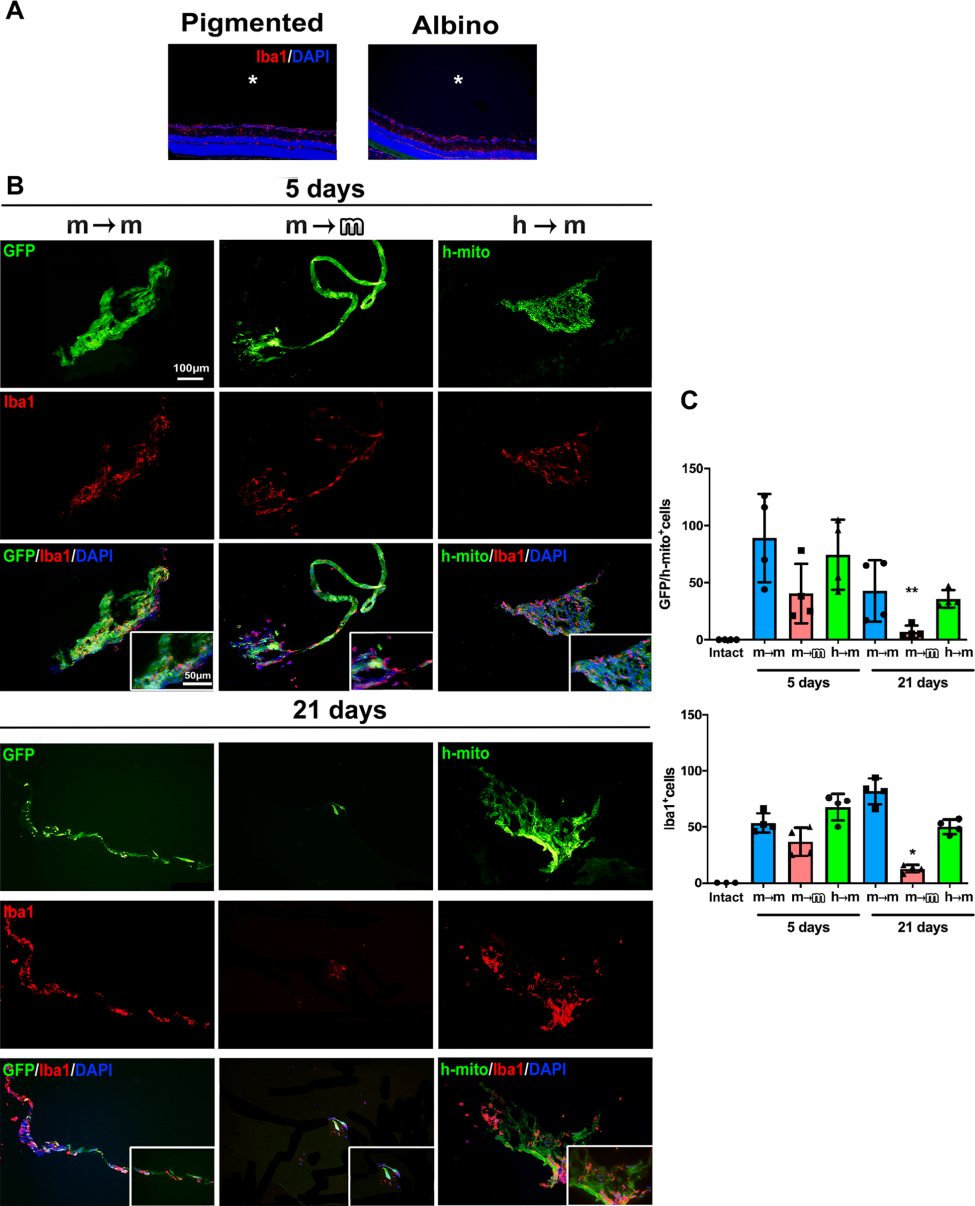
Microglial cells migrate to the vitreous and surround BM-MSC transplants. **A** In intact albino and pigmented mice, microglial cells (red) were restricted to the retina, while the vitreous (asterisks) was cell free, as there was neither DAPI-stained nuclei nor Iba1 signal. **B** At 5 and 21 days after the transplant, BM-MSCs were found in the vitreous forming a meshwork (green, GFP signal in mouse to mouse and human mitochondrial (h-mito) immunodetection in human to mouse). In all transplantation settings, microglial cells (red) migrated to the vitreous and surrounded the transplanted cells. **C** Column graphs showing the mean number per section and animal ± SD of transplanted cells (top) or microglial cells (bottom) quantified in the vitreous of intact animals and at 5 and 21 days after each transplant (*p < 0.05 comparing 21 and 5 days, nonparametric Mann–Whitney *t* test). *n* = 4 retinas/group/timepoint, 3 sections/retina

The vitreous of intact retinas was free of microglial cells but in the transplanted retinas, the presence of BM-MSCs in the vitreous attracted Iba1^+^ microglial cells. Within transplants and between 5 and 21 days, significant changes were observed only for the allotransplant, with a decrease of microglial cells and BM-MSCs numbers. Although there was a decrease of BM-MSCs at 21 days in the other two transplantations settings it did not reach significance (Fig. 5).

These data indicate that the cells remain in the vitreous for up to 21 days, without integrating into the retina, and are being cleared by microglial cells, a clearance that is quicker in allogenic transplants.

### Immunosuppression in allotransplants

To recreate the most common clinical settings, two groups of animals were immunosuppressed for the duration of the procedure right after allogeneic transplantation of BM-MSCs. These groups were independent since to carry out the OCT and ERG analyses, animals had to be euthanised immediately after these techniques (5 days and 21 days). For this reason, we omitted 3 days to save animals and because there were no functional or anatomical changes between 3 and 5 days in any transplant modality.

**Fig. 6:**
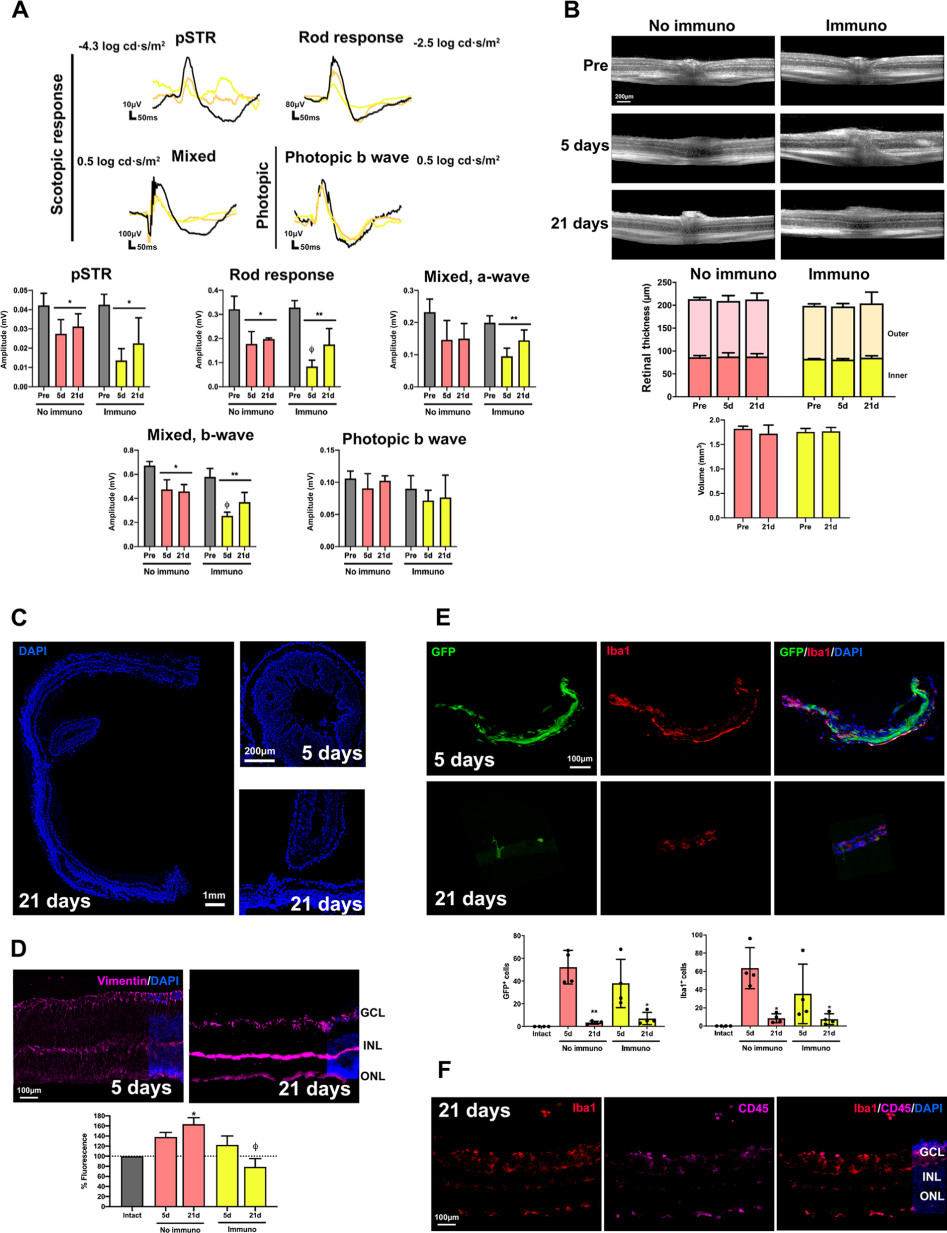
Systemic immunosuppression in allotransplanted animals further impairs retinal functionality and decreases Müller cell activation and microglial activation. **A** Electroretinogram waves obtained from the scotopic and photopic response before (pre-, black) and at 5 and 21 days in allotransplanted retinas from immunosuppressed animals (non-immunosuppressed waves are shown in Fig. 2) and below the quantification graphs (mean wave amplitude ± SD). *Significant compared to baseline values (*p < 0.05, **p < 0.01, nonparametric multiple comparisons Kruskal–Wallis' test and Dunn’s post hoc analysis). ^Ɵ^Significant comparing non-immunosuppressed with immunosuppressed animals (^Ɵ^*p* < 0.05, nonparametric *t* test, Mann–Whitney's). **B** Top, OCT sections spanning the optic nerve taken before (pre-) and at 5 and 21 days in allotransplanted retinas from non-immunosuppressed and immunosuppressed animals. Bottom, measurement of OCT images: stacked column graphs showing the total, outer and inner retinal thickness measured at 1-mm from the optic nerve (mean ± SD) and column graph showing the central retinal volume. **C** DAPI-stained cross sections from immunosuppressed retinas analysed at 5 and 21 days showing alterations in the retinal architecture. **D** Retinal cross sections showing Müller cells (vimentin, purple) in intact and allotransplanted retinas from non-immunosuppressed and immunosuppressed animals 5 and 21 days after the transplant and quantification of fluorescence intensity relative to intact retinas. *Significant comparing with intact, ^Ɵ^Significant comparing non-immunosuppressed with immunosuppressed (^Ɵ^*p* < 0.05, nonparametric *t* test, Mann–Whitney's). **E** GFP^+^ BM-MSCs in the vitreous of allotransplanted retinas with immunosuppression and column graphs showing the mean number per section and animal ± SD of transplanted cells (left) or microglial cells (right) quantified in the vitreous of intact animals and at 5 and 21 days in allotransplanted retinas with or without immunosuppression (*p < 0.05 comparing 21 and 5 days, nonparametric Mann–Whitney *t* test). **F** Retinal magnifications showing Iba1 (red) and CD45 (purple) expression in the immunosuppressed + allotransplants group. *n* = 4 retinas/group/timepoint, 3 sections/retina

Immunosuppression impaired at 5 days the retinal functionality further than allotransplant alone for the rod response and the mixed a-wave response (Fig. 6A). This higher functional impairment was transient and at 21 days these waves reached amplitudes similar to allotransplant without immunosuppression. Immunosuppression + allotransplantation did cause retinal abnormalities that were not seen in vivo (Fig. 6B) but were visible in cross sections (Fig. 6C) and with the same frequency (25% of retinas) than allotransplantation alone.

When we analysed Müller cell hypertrophy, we observed that 21 days of immunosuppression significantly reduced the expression of vimentin compared to what was observed in allotransplants (Fig. 6D). Neither functional, anatomical nor glial alterations were observed in immunosuppressed control animals (immunosuppression without transplant, Additional file 3: Fig. S3).

Migration of microglial cells into the vitreous and survival of the transplanted cells was similar between allotransplants with or without immunosuppression (Fig. 6E). Interestingly, microglial cells in the retina did not show anatomical features of activation in the immunosuppressed group (Fig. 6F), and indeed, in these retinas the percentage of CD45^+^ cells was similar to that found in intact ones (0.30%).

### BM-MSC transplantation changes the profile of pro- and anti-inflammatory molecules

**Fig. 7:**
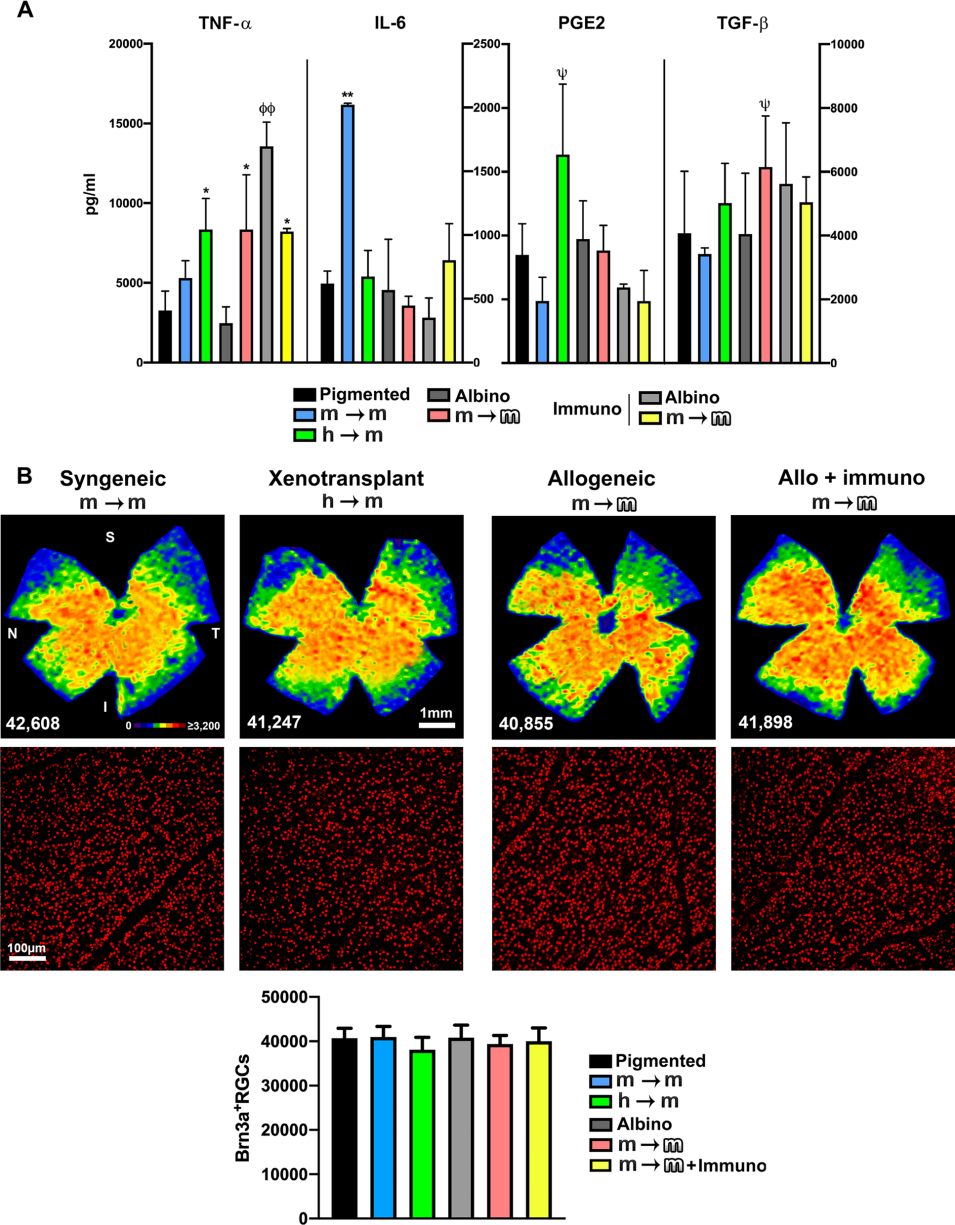
BM-MSC transplants alter the profile of pro- and anti-inflammatory cytokines in the retina, but do not cause retinal ganglion cell death. **A** Column graphs showing the mean concentration (pg/ml) ± SD of tumour necrosis factor α (TNF-α), interleukin 6 (IL-6), prostaglandin E2 (PGE2) and transforming growth factor β (TGFβ) in retinal extracts from all groups analysed at 21 days after each transplant. *Significant compared to intact values (*p < 0.05, **p < 0.01), ^Ɵ^Significant comparing non-immunosuppressed with immunosuppressed (^Ɵ^*p* < 0.05), ^ψ^Significant comparing xeno- and allotransplants with syngeneic transplants (^ψ^*p* < 0.05). Nonparametric Mann–Whitney *t* test. *n* = 4 retinas/group and 3 replicates/plate. **B**: Top: isodensity maps showing the topography of RGCs in transplanted retinas analysed 21 days after each transplant. Below each map is shown the total number of RGCs counted in its corresponding retina. Middle: magnifications showing Brn3a^+^RGCs in flatmounts. Bottom: column graph showing the mean total number of RGCs ± SD in each group. *n* = 6 animals/group

In retinal extracts, we measured the levels of two proinflammatory (TNF-α and IL-6) and two anti-inflammatory (PGE2 and TGF-β) molecules (Fig. 7A). TNF-α levels in the retina were significantly higher in allo- and xenotransplants than in their corresponding intact controls. Surprisingly, in immunosuppressed animals without transplant, TNF-α levels were threefold higher than those found in non-immunosuppressed mice. Immunosuppressed and allogenically transplanted retinas showed a significant increase of this cytokine compared to intact controls, but a decrease compared to immunosuppressed controls.

Regarding IL-6 levels, the only significant change was observed in syngeneic transplants, where the concentration of this cytokine increased significantly to threefold that of intact animals.

Although there were some fluctuations, anti-inflammatory molecules did not significantly change compared to intact retinas. However, the concentrations of PGE2 in xenotransplanted and TGF-β in allotransplanted retinas were significantly higher than in syngeneically transplanted retinas.

### Intravitreal transplantation of BM-MSCs does not trigger retinal ganglion cell (RGC) death

Because allo- and xenotransplants caused intense microglial activation in the ganglion cell layer, and a reduction of RGC functionality, we wondered whether these changes were detrimental to these neurons. Thus, we quantified their total population in intact and transplanted retinas using as marker Brn3a, which is expressed in viable RGCs [7]. Our results show that in spite of all the retinal changes triggered by the cells, none of them caused RGC loss up to 21 days (Fig. 7B and Additional file 2: S2C).

## Discussion

Systematic and comparative studies are essential to properly gauge the therapeutic outcome of cell therapy and thus to translate preclinical studies to patients. The misleading concept of the immune privileged status of the central nervous system together with the immunomodulatory properties of MSCs, has led in the past to administer these and other cells into the nervous tissue without paying proper attention to the host response. With few exceptions [14, 37] most preclinical studies analyse cell efficacy against a variety of insults or diseases [14, 18, 36] disregarding the effect of cell transplantation itself in healthy systems.

Previously, we showed that the syngeneic intravitreal transplant of BM-MSCs was neuroprotective and promoted axonal regeneration of axotomized RGCs, while allogeneic transplant did not differ from un-transplanted retinas. Xenogeneic transplant, however, impaired retinal functionality further than axotomy alone [18]. Those data tie beautifully with data presented here, where we show that in the healthy retina, the syngeneic transplants neither alter retinal function nor cause glial activation, while allo- and xenotransplants do, being the latter the most damaging transplant.

Hwang et al. [37] described the differential glial activation in the mouse brain when transplanting adipose MSCs in xenogeneic, allogeneic and syngeneic mode. In agreement with us, they observed a higher number of CD45^+^cells, leukocytes and neutrophils in the xenotransplanted samples followed by allotransplanted and these by syngeneically transplanted ones. This indicates that there is a common response of the CNS to MSC administration, regardless of MSC provenance and injection site [38, 39].

Transplanted BM-MSCs are found in the vitreous without integrating in the retina. Their number decreases from early time points to 21 days, and at 3 months post-transplant, they have disappeared, as we showed before [18]. Loss of MSCs after transplantation is a common feature [14, 37, 40] that concords well the microglial/macrophage activation observed here and in other reports [14, 37]. The most toxic environment for BM-MSCs is not xenogeneic as expected, but allogeneic. Human BM-MSCs are highly immunosuppressive [41] which together with their specific immunomodulatory mechanisms that differ in some respects from those reported for mouse [42] could explain why hBM-MSCs survive better than mBM-MSCs in allogeneic transplants.

We anticipated a higher BM-MSC survival in the immunosuppressed group and although immunosuppression maintained CD45 signal at intact levels in the allotransplanted retinas, it did not damp microglial migration to the vitreous. Cyclosporine inhibits T-cell and the adaptative immune response, so here it may not influence. Cortisone, on the other hand, affects the innate immune cells. Low doses of cortisone (< 5 mg/kg) stimulate the phagocytic activity of macrophages [43] and CD45^low^ microglial cells express phagoptosis genes [44], both may explain the poor BM-MSC survival on immunosuppressed animals.

Retinal remodelling was observed in vivo only in xenotransplanted retinas. Ex vivo, however, retinal folds were seen in all transplantations with a different prevalence and location. This in vivo–ex vivo difference is a technical matter because OCT captures the central retina and the remodelling may be peripheral, depending on the proximity of the transplanted cells.

Reactive gliosis and microglial activation occur in response to stress or damage [45–47], and it was observed in all transplants in the areas of retinal abnormalities. Importantly, Müller cell gliosis and microglial activation did spread to the rest of the retina after allo- and xenotransplants but not after syngeneic transplantation.

Hypertrophy causes Müller cell stiffness, which is believed to serve as a scaffold for microglial cells [48], and it has been associated with architectural changes, such as the ones observed here [49]. Iba1^+^ and/or CD45^+^ cells are present in the retinal folds which may also exacerbate these abnormalities. Furthermore, Müller cells secrete TNF-α, which in turn increases the expression of inflammatory mediators, such as IL-6 that promote microglia/ monocyte infiltration into the retina [50]. TNF-α, is upregulated in xeno and allotransplanted retinas and IL-6 in syngeneically transplanted ones. Activation of microglial and Müller cells by these cytokines may be part of the complex host–graft response observed in all transplants. However, the differential cytokine profile and the different frequency of abnormalities as well as the fact that xenotransplants induce the strongest response with recruitment of CD45^+^ cells, indicates that there are factors yet unknown which require further investigation if we aim to understand and control these pathological events.

Functional impairment was observed after allo- and xenotransplants, but not after syngeneic ones. Microglial cells in allogeneic and xenogeneic transplants are activated, and proinflammatory microglia releases miRNAs that decrease excitatory synapsis [51]. In addition, it has been reported that microglia modulate retinal function by secreting complement molecules that are essential for the synapse pruning carried out by microglial cells in development and homeostasis [52, 53]. Over-secretion of these factors by activated microglial cells would lead to an inadequate synapse targeting and pruning which in turn would affect function. Functional impairment may also be related to Müller cell hypertrophy. Although Müller cell contribution to the ERG waves is controversial in terms of strength [54], it is accepted that they influence the ERG output. And so, here we see that in those situations with Müller cell hypertrophy the retinal function is impaired. In immunosuppressed and allotransplanted retinas, the b-wave and rod response wave were lower than in allotransplanted ones at 5 days, recovering at 21 when Müller cell gliosis was reduced below intact levels.

Syngeneically transplanted retinas do not show widespread Müller or microglial activation and differ from the other groups in the overexpression of IL-6. IL-6 is a pleiotropic cytokine with anti- and proinflammatory properties which plays important regulatory roles in the adult central nervous system, with both beneficial and harmful roles, depending on the context. And thus, it can promote neuronal survival after injury or cause neuronal death in neurodegenerative disorders. The neuroprotective role of IL-6 goes through modulation of excitability and function by regulating voltage-gated and receptor operated channels, which in turn are critical for neuronal electrical functionality (reviewed in [55–57]). Here, levels of IL-6 three times higher than basal conditions did not cause RGC death, and thus, it is tempting to speculate that in this context IL-6 is not detrimental but beneficial, which concords with the maintenance of functionality seen here and the neuroprotective and neuroregenerative properties of syngeneic BM-MSCs [18].

One striking result was the strong upregulation of TNF-α in immunosuppressed retinas, which was partially controlled by the allotransplant. Corticosteroid treatments decrease TNF-α serum levels. However, different tissues in different circumstances might respond differently, as it has been reported for endothelial cells [58] and we show here in the retina. At present we do not know the significance of TNF-α overexpression in otherwise healthy retinas, but it is important to realize that the high levels of TNF-α in immunosuppressed retinas did not cause functional impairment, retinal gliosis or RGC death, indicating that this cytokine in these conditions does not activate glial cells and is not per se damaging to neurons.

Our data here point to syngeneic transplants being the safest for clinical translation. Indeed, syngeneic, but not xenogeneic, transplants rescue both RGCs [18] and photoreceptors [59, 60]. However, syngeneic (autologous) transplants in the clinic may not always be feasible due to ageing or underlying patient pathologies that may exhaust MSCs rendering them ineffective. To solve this problem, induced pluripotent stem cells could be the next step, but their preparation and proper differentiation into MSCs is laborious at the moment. However, the establishment and exploitation of standardized MSC biobanks would be an interesting alternative to increase the chances of finding compatible donors, despite further research being needed to investigate the influence of such immune-compatible allogeneic donor MSCs on retinal functioning.

Despite all the functional, glial and cytokine changes induced by the transplanted cells and the enormous anatomical remodelling observed, especially in xenotransplants, the population of RGCs remained within intact values. This brings hope to BM-MSC-based therapies in the CNS, because neuronal death is irreversible, but with greater knowledge and understanding of the host–graft cross talk we can design more efficient strategies to modulate damaging responses and enhance beneficial ones.

## Conclusions

**Fig. 8:**
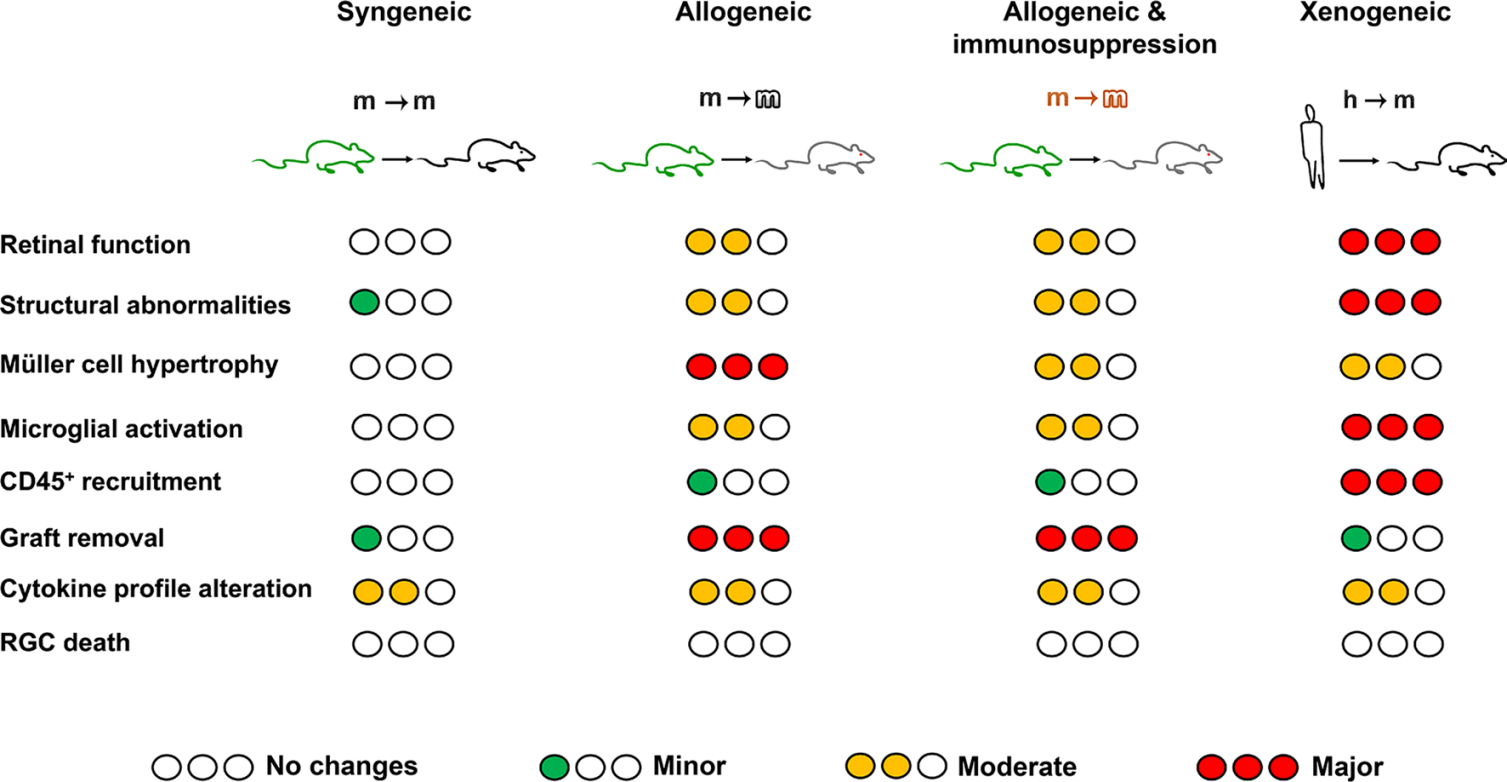
Graphical summary of the effects of BM-MSC transplantation type on the healthy retina. Syngeneic transplants induce fewer changes in the retina than allotransplants or allotransplant with immunosuppression (orange) and these than xenotransplants. Recruitment of CD45^+^cells and microglial and Müller cell activation refer to the whole retina

Here we show how BM-MSCs transplantation affects the host tissue (Fig. 8) and how this bidirectional (host–graft) effect varies according to the type of transplant. As expected, xenotransplant are the more damaging, followed by allotransplants with or without immunosuppression. Syngeneically administered cells are not completely innocuous, but they do not alter neuronal functionality, which makes them the safest and in agreement with [18, 59, 60] the best for neuroprotection and induction of axonal regeneration. If we want to safely and successfully transfer cell therapy to patients with neurodegenerative diseases, systematic and comparative works are therefore essential, as much research remains to be done to decipher the intricate interaction between graft and host.

## Supplementary Information

**Additional file 1: Fig. S1.** Mouse and human MSC immunophenotype and multilineage differentiation properties. **A** Bone marrow MSCs from human and mouse were analysed for the expression of the MSC surface markers CD73, CD90 and CD105, and the haematopoietic markers CD14, CD20, CD45 and CD34 by flow cytometry. Control isotypes staining (grey histograms) are shown. **B** hBM-MSCs and mBM-MSCs were cultured in adipogenic, osteogenic and chondrogenic differentiation media to evaluate their multilineage differentiation properties. Adipogenic differentiation was assessed by lipid droplets staining using Oil Red O solution. Osteogenic differentiation was evaluated by detecting calcium deposition and alkaline phosphatase activity by Alizarin Red and BCIP/NBT staining, respectively. Finally, chondrogenic differentiation was evaluated by detecting expression of glycosaminoglycans by Alcian blue and eosin staining. Scale bar: 200 µm.

**Additional file 2: Fig. S2.** Anatomy in intact and vehicle-injected retinas, and microglial cells in allotransplants. **A** DAPI-stained retinal cross sections from intact albino and pigmented mice and a vehicle-injected pigmented mice processed 5 days after the injection. **B** Immunodetection of Brn3a and CD45 in a retinal cross section from an animal analysed 21 days after allotransplant. CD45^+^cells are observed in the retinal fibre layer above the RGCs (Brn3a^+^). **C:** RGC isodensity maps from intact retinas.

**Additional file 3: Fig. S3.** Immunosuppression alone does not alter the anatomy, glial status or function of the retina. **A** Graphs showing the mean wave amplitude ± SD of the electroretinographic waves in intact (grey bars) and systemically immunosuppressed animals (orange bars). **B** Representative DAPI-stained retinal section from an immunosuppressed animal. **C** Retinal cross section showing Müller cells (vimentin, purple) in immunosuppressed animals and quantification of fluorescence intensity relative to intact retinas (grey bars, 100%). *n* = 4 retinas/group.

## Data Availability

Supplementary material is available at stemcellres.biomedcentral.com.

## Abbreviations

BM-MSC: Bone Marrow-derived Mesenchymal Stromal Cell
DMEM: Dulbecco’s modified Eagle’s medium
ERG: Electroretinography
FBS: Foetal Bovine Serum
GFP: Green Fluorescent Protein
MSC: Mesenchymal Stromal Cell
OCT: Optic Coherence Tomography
PGE2: Prostaglandin E2
pSTR: Positive Scotopic Threshold Response
RGC: Retinal Ganglion Cell
SD: Standard Desviation
TGFβ: Transforming Growth Factor beta
TNF-α: Tumour Necrosis Factor alpha
