# Supplementary figures and images for: Immune recognition of syngeneic, allogeneic and xenogeneic stromal cell transplants in healthy retinas
Source: Stem Cell Res Ther. 2022 Aug 20;13:430. doi: 10.1186/s13287-022-03129-y (PMC9392272; doi:10.1186/s13287-022-03129-y)

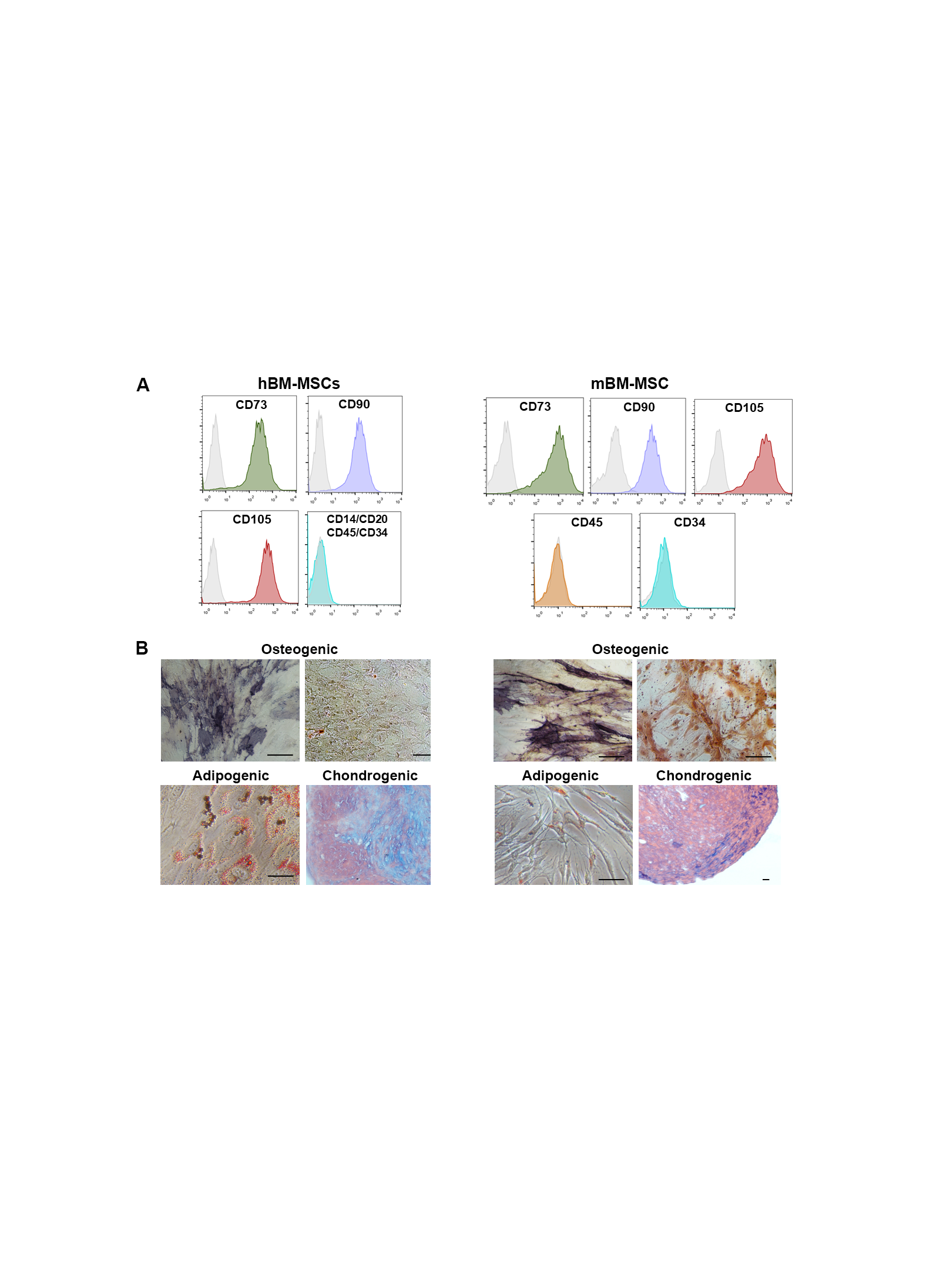

Supplement: Supplementary file 1 — Additional file 1: Fig. S1. Mouse and human MSC immunophenotype and multilineage differentiation properties. A Bone marrow MSCs from human and mouse were analysed for the expression of the MSC surface markers CD73, CD90 and CD105, and the haematopoietic markers CD14, CD20, CD45 and CD34 by flow cytometry. Control isotypes staining (grey histograms) are shown. B hBM-MSCs and mBM-MSCs were cultured in adipogenic, osteogenic and chondrogenic differentiation media to evaluate their multilineage differentiation properties. Adipogenic differentiation was assessed by lipid droplets staining using Oil Red O solution. Osteogenic differentiation was evaluated by detecting calcium deposition and alkaline phosphatase activity by Alizarin Red and BCIP/NBT staining, respectively. Finally, chondrogenic differentiation was evaluated by detecting expression of glycosaminoglycans by Alcian blue and eosin staining. Scale bar: 200 µm. [file 13287_2022_3129_MOESM1_ESM.tif]

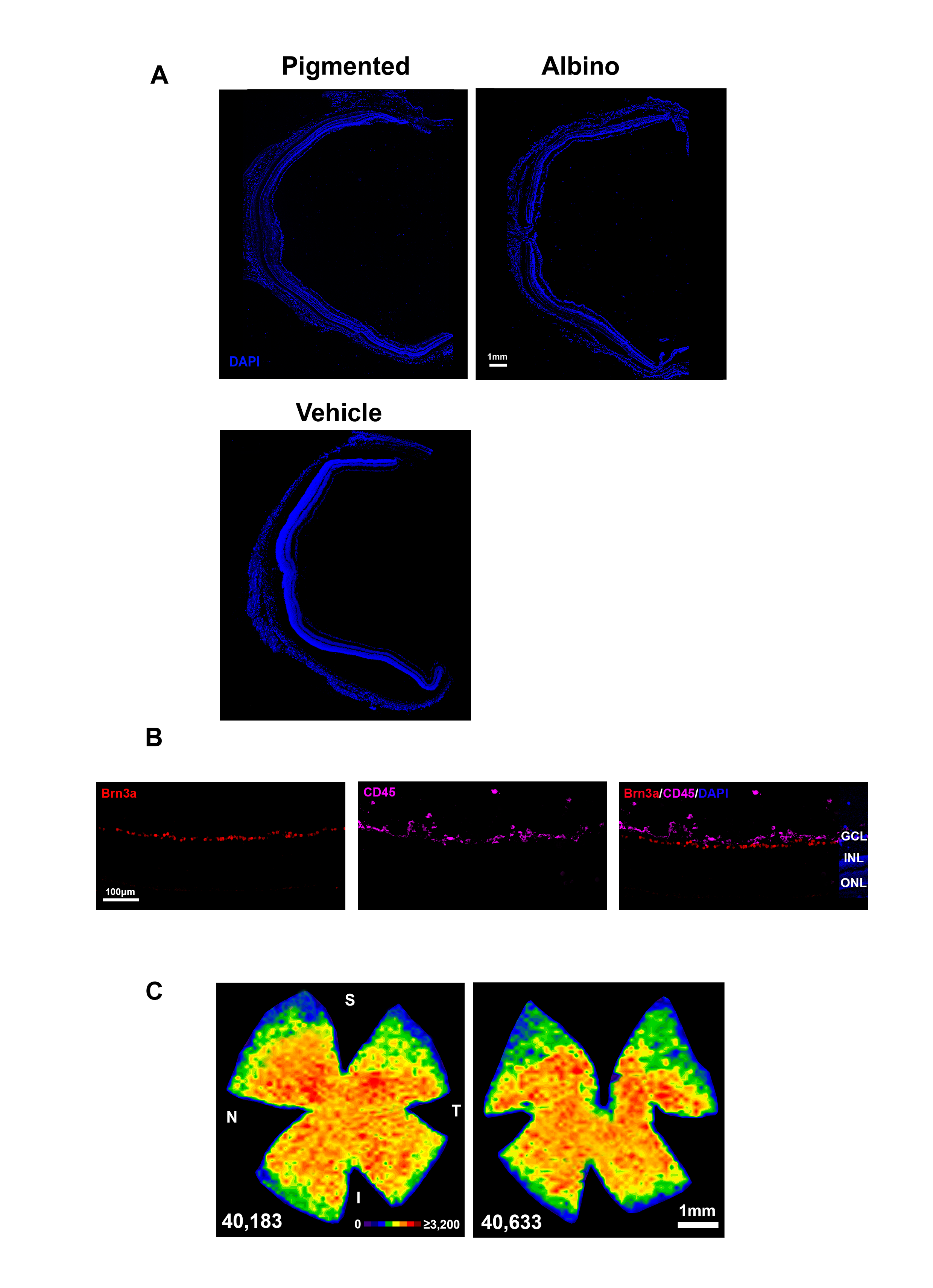

Supplement: Supplementary file 2 — Additional file 2: Fig. S2. Anatomy in intact and vehicle-injected retinas, and microglial cells in allotransplants. A DAPI-stained retinal cross sections from intact albino and pigmented mice and a vehicle-injected pigmented mice processed 5 days after the injection. B Immunodetection of Brn3a and CD45 in a retinal cross section from an animal analysed 21 days after allotransplant. CD45+cells are observed in the retinal fibre layer above the RGCs (Brn3a+). C: RGC isodensity maps from intact retinas. [file 13287_2022_3129_MOESM2_ESM.tif]

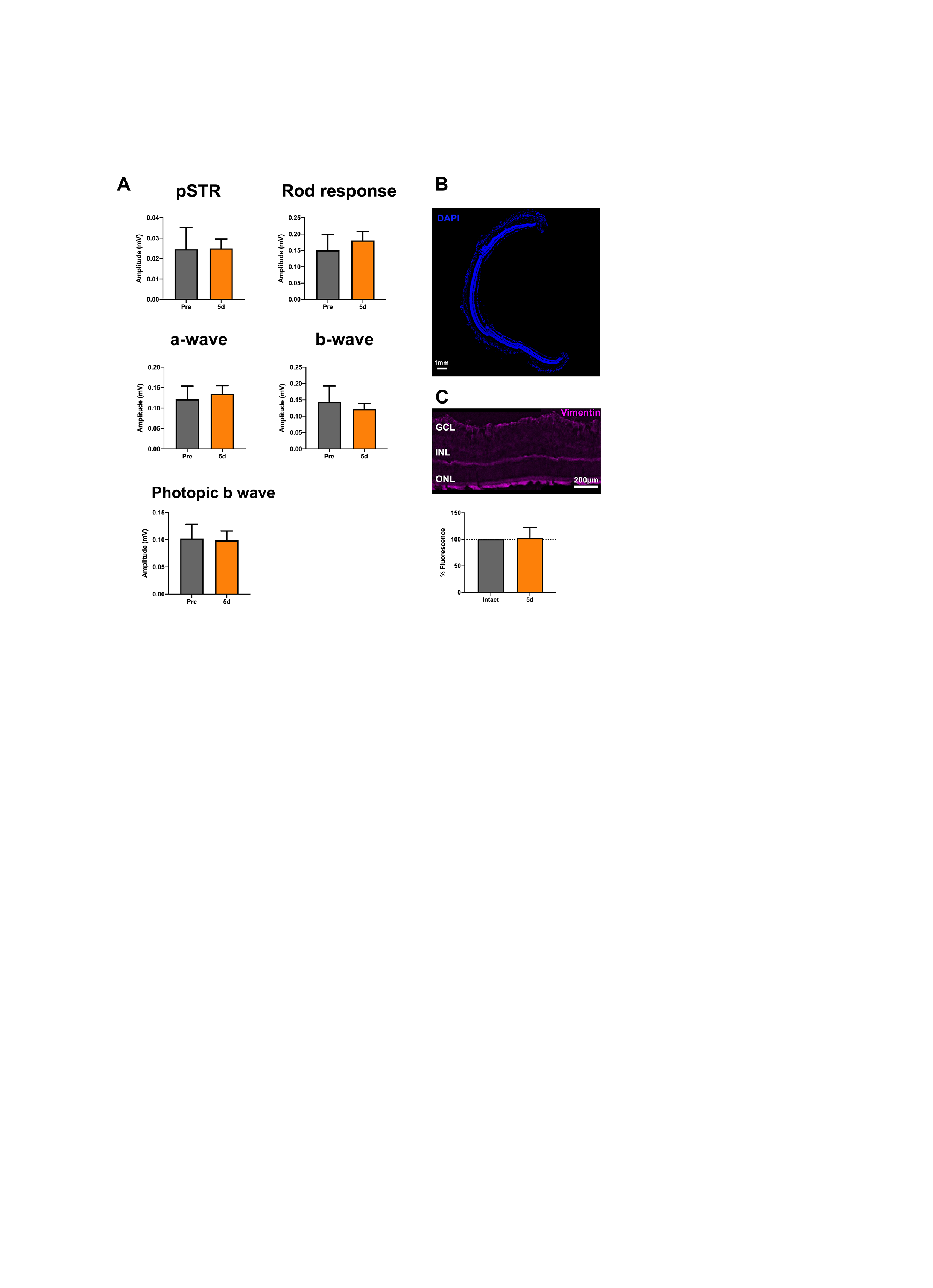

Supplement: Supplementary file 3 — Additional file 3: Fig. S3. Immunosuppression alone does not alter the anatomy, glial status or function of the retina. A Graphs showing the mean wave amplitude ± SD of the electroretinographic waves in intact (grey bars) and systemically immunosuppressed animals (orange bars). B Representative DAPI-stained retinal section from an immunosuppressed animal. C Retinal cross section showing Müller cells (vimentin, purple) in immunosuppressed animals and quantification of fluorescence intensity relative to intact retinas (grey bars, 100%). n = 4 retinas/group. [file 13287_2022_3129_MOESM3_ESM.tif]
